# Prevalence and Risk Factors of Irritable Bowel Syndrome Among Adults in Al-Qunfudah Governorate, Saudi Arabia

**DOI:** 10.7759/cureus.48639

**Published:** 2023-11-11

**Authors:** Safa H Alkalash, Rahmah A Almagadi, Shathah M Alamri, Layla A Al-amri, Mashael A Al-amri, Jawaher M Al-amri, Maryam H Almaqadi

**Affiliations:** 1 Community Medicine and Health Care, Umm Al-Qura University, Al-Qunfudah, SAU; 2 Family Medicine, Menoufia University, Shebin Elkom, EGY; 3 Al-Qunfudah College of Medicine, Umm Al-Qura University, Al-Qunfudah, SAU

**Keywords:** rome iv, quality of life, gad-7, generalized anxiety disorder, ibs, irritable bowel syndrome

## Abstract

Background

Irritable bowel syndrome (IBS) is the most prevalent functional gastrointestinal disorder and is linked to numerous psychiatric abnormalities.

Objectives

In Al-Qunfudah governorate, this study was done to estimate the prevalence of IBS and its associated risk factors among adults.

Material and methods

The Rome IV criteria and GAD-7 (General Anxiety Disorder-7) were used in this community-based cross-sectional study. A total of 335 adults from the general population made up the sample, which was chosen using a convenience (non-probability) sampling technique. The required data were collected through the dissemination of the survey link online through different electronic platforms.

Results

The total prevalence of IBS was 30.4% (n = 102), with females having a greater prevalence (55.9%, n = 57 out of 102). GAD was found in 15.8% of the total participants (n = 53). Significant relationships were discovered between IBS and marital status, education, occupation, smoking status, IBS history, and anxiety levels (p-values were <0.001, 0.023, 0.006, 0.016, <0.001, and <0.001, respectively). According to regression analysis, being single, having a family history of IBS, and having a generalized anxiety disorder were all risk factors for IBS.

Conclusion

In this study, the prevalence of IBS among adults in Al-Qunfudah governorate, Saudi Arabia, was shown to be 30.4%. Further, approximately 16% of individuals had generalized anxiety disorder. Being unmarried, having a positive family history of IBS, and suffering from GAD were all risk factors for developing IBS. People need to be educated on the symptoms and consequences of IBS. We also propose that those suffering from IBS symptoms seek a second opinion from a doctor to manage this problem and its impact on their quality of life.

## Introduction

One of the most common chronic gastrointestinal illnesses is irritable bowel syndrome (IBS). It is characterized by frequent abdominal pain associated with changes in the stool appearance or frequency in the absence of obvious anatomical or physiological abnormalities. Most people's signs and symptoms worsen over time, but occasionally they improve [1, 2]. The American College of Gastroenterology IBS Task Force defined IBS as abdominal pain or discomfort that occurs in association with altered bowel habits over a period of at least three months [3]. Changes in bowel function, with the primary bowel symptom establishing IBS sub-classification: IBS with constipation (IBS-C), IBS with diarrhea (IBS-D), or IBS with constipation and diarrhea alternating (IBS-M) [4]. Irritable bowel syndrome (IBS) was described by Rome IV as a functional bowel condition characterized by recurring abdominal pain relevant to defecation or a change in bowel habits. Disordered bowel habits are typically present (i.e., constipation, diarrhea, or a mix of constipation and diarrhea), as are symptoms of abdominal bloating or distension. Symptom onset should occur at least six months prior to diagnosis, and symptoms should be present during the last three months [5].

IBS affects at least 7-21% of the adult population globally, with a considerable female predominance and being more prevalent in people under 50 [6]. In a recent Saudi study, the reported prevalence rate for IBS in the south-west area of Saudi Arabia was 23.8% and was associated with being female, a tobacco smoker, and having mental health issues. [7]. IBS can affect a broad spectrum of ages and economic, social, and ethnic backgrounds [6]. Furthermore, there are varieties of primarily recorded risk factors, such as changes in gastrointestinal tract motility, an abnormal nervous system, hormones, severe infection, changes in gut microbes, genetic causes, which are rare, and non-allergic dietary factors [8].

There are several IBS medications, including those for constipation (IBS-C), diarrhea (IBS-D), and mixed symptoms (IBS-M). This main category of drugs is divided into three subcategories: therapies for common symptoms, newer treatments, and alternative treatments [9]. Treatment for sup-type IBS-D typically begins with antidiarrheals, then antidepressants, then antispasmodics, and finally an osmotic laxative. Lubiprostone and linaclotide are two treatments for the condition (IBS-C). Alternative treatments for the IBS-M subtype include placebo effects, peppermint oil, and probiotics [10]. The complications of IBS are as follows. Hemorrhoids can develop because of chronic diarrhea or constipation. Furthermore, many people with mild to severe IBS have a low quality of life, which necessitates frequent hospital visits and costly investigations [11]. According to research, individuals with IBS reported missing an average of 13.4 days of work or school per year, compared to 4.9 days for those without IBS, according to a survey addressed to US households [12].

Generalized anxiety disorder (GAD) is characterized by persistent and excessive worry that disrupts ordinary activities. This constant anxiety and stress may be accompanied by physical manifestations such as restlessness, feeling tense or easily exhausted, difficulties staying focused, tension in the muscles, or difficulty drifting off to sleep. Worries about ordinary things like work commitments, the health of the family, or little issues like chores, car maintenance, or appointments are common [13]. Anxiety has been associated with the etiology and severity of irritable bowel syndrome. Several studies have revealed that patients with IBS have a significant frequency of psychiatric illnesses, and symptomatology should be routinely assessed and treated since psychological factors are significant moderators of symptom severity, symptom persistence, decision to seek treatment, and response to treatment [14-16]. Disruption of the bidirectional brain-gut axis is becoming more widely accepted as a conceptual model of IBS pathogenesis, incorporating inappropriate function in the enteric, autonomic, and/or central neurological systems [17]. Additionally, IBS symptoms can cause mood disorders, which can exacerbate depression or anxiety. Despite not being a life-threatening disease, IBS has a significant economic impact in comparison to healthy people [11]. Thus, the purpose of this study was to estimate the prevalence and risk factors of irritable bowel syndrome in adults in Al-Qunfudah governorate, Saudi Arabia, because this governorate is regarded as a remote Saudi location that has limited health care services and no studies on IBS have been conducted in this area.

## Materials and methods

Study design

This community-based cross-sectional study was conducted among Al-Qunfudah adult citizens (age ≥18 years) of both male and female genders during a period of three months from January to March 2023, and data were collected through an electronic survey that was disseminated via different electronic platforms. Adults who self-reported experiencing any of the following conditions-chronic fever, bloody diarrhea, or progressive unexplained weight loss-as well as those who declined to provide written informed consent for participation were excluded from the study.

Study setting

Al-Qunfudah governorate, a province of Makkah, was selected purposefully as the study location. The governorate of Al-Qunfudah is located in the Tihamah plain on Saudi Arabia's Red Sea coast. It is the fourth-largest populated province in the Makkah Region and faces the Red Sea to the west. It is 350 kilometers to the south of Makkah and 360 kilometers to the south of Jeddah. Its location on a map is 19.1281° North, 41.0787° East. 

Sample size

The data were collected from a convenience sample of adults who were residents of Al-Qunfudah governorate at the time of data collection. The sample size was calculated using EPI Info (United States Centers for Disease Control, Atlanta, USA). Based on the total population number in the Al-Qunfudah governorate of 300,516 and the prevalence of IBS of 24% [7], with a confidence interval of 95% and a margin of error of 5%, the minimum calculated sample size was 280 participants.

Tools for data collection

An online survey was created via the Google form, and this process has been done in many phases. Firstly, a focused literature review was done, followed by a selection of the relevant information, and then the survey items were created and drafted as a 23-item Arabic survey. Its items were organized and reviewed by a panel of experts (family medicine and internal medicine) who assessed the relevance of each item to the research topic, and they developed consensus on the selected sociodemographic items and an opening question of whether the participants had been diagnosed with IBS. Furthermore, they recommended using Rome IV and GAD-7 because they are validated questionnaires. Finally, it was pre-tested through the application of a pilot study. The main target of this pilot was to assess the relevance of the designed survey before its use, to determine whether it would be understandable by people with a wide variety of educational backgrounds and experiences, the time required to fill it out, and the response rates. The survey link was disseminated electronically via WhatsApp, Telegram, and Snapchat, and the participants were invited to fill out this survey voluntarily. The first 40 submitted answers were collected; then, the survey link was closed until the collected data had been analyzed. Finally, the reliability of the survey was evaluated using the test-retest technique. Cronbach’s alpha coefficient was 0.78.

The final form of the applied survey was composed of 23 questions and distributed into three sections. The first section consisted of seven items to assess sociodemographic data such as age, gender, social status, education level, job status, and smoking. The second section consisted of ten items. Firstly, to assess whether respondents had been diagnosed with IBS. Secondly, a set of diagnostic standards known as the Rome IV criteria [5] was used to recognize and categorize functional gastrointestinal disorders (FGIDs). The criteria are accepted as the gold standard for diagnosing FGIDs and have been developed by a team of internationally recognized gastrointestinal experts. Based on the main symptoms, such as abdominal discomfort, bloating, and abnormal bowel habits, the criteria divide FGIDs into a few categories. A person must have had recurrent abdominal pain or discomfort for at least three days each month for the previous three months to meet the Rome IV criteria for irritable bowel syndrome (IBS). This pain or discomfort must be linked to two or more of the following: improvement with defecation, onset linked to a change in frequency of stool, and onset linked to a change in form (appearance) of stool. Thirdly, to assess whether the respondents were taking any medication for their symptoms, the influence of the symptoms on their lives, their family history of IBS, and the relationship between their symptoms and food. The final section of the survey included the Arabic version of the GAD-7 questionnaire [18] to evaluate the prevalence of GAD among the study group because it is likely that both GAD and IBS involve disruptions of the central and peripheral serotonergic systems. Additionally, they cause avoidance behaviors and anticipatory anxiety that make it difficult to operate in daily life. GAD-7 consists of seven questions about anxiety, such as feeling tense, nervous, or on edge; not being able to stop or control worrying; worrying excessively about various issues; having difficulty relaxing; being so restless that it's difficult to sit still; easily becoming irritated or annoyed; and feeling afraid that something terrible might happen. For all questions, answer with one of the following: Not at all = 0, several days = +1, more than half the days = +2, and nearly every day = +3. The total score ranged from 0 to 21 and was subdivided into three categories: 0-4 points is minimal anxiety; 5-9 points means mild symptoms; 10-14 points refers to moderate symptoms; and more than 15 points denotes severe symptoms. The optimal cut-off point in this scale is 10, therefore a score of less than 10 indicates low anxiety, whereas a score of 10 or more indicates moderate to high anxiety (likely anxiety). 

Procedure of data collection

The survey link was distributed on a number of digital channels, including WhatsApp, Telegram, Twitter, and Snapchat, because these platforms have a particularly high rate of usage among the target population. As a result, the required study data were gathered over the course of three months, from January to March 2023. The survey inquired about each participant's place of residence in order to verify that all data came from the Al-Qunfudah adult population; those who answered otherwise had been excluded and were not included in the study's final conclusions. After sorting through the 354 responses we got, we discovered that eight of them were incomplete and that 11 of them originated from outside the governorate of Al-Qunfudah. Both were excluded from the study of the data. In the end, 335 surveys were approved to be analyzed.

Ethical considerations

The Medical Research and Ethics Committee of the College of Medicine, Umm Al-Qura University, Makkah, Kingdom of Saudi Arabia, granted this study ethical permission and assigned it approval number HAPO-02-K-012-2023-02-1427. The questionnaire began with an opening question designed to elicit consent from all participants while ensuring that the data collected would be kept confidential. No participant-specific personal information was gathered, and all information was tagged and handled with care to guarantee its security. Access to the research data is restricted to the main investigator and other assigned researchers, and it is securely saved on a portable external drive.

Data analysis

The data were analyzed statistically using SPSS version 26 (IBM Corp., Armonk, USA). Frequencies and percentages were used for expressing qualitative data, whereas the mean and standard deviation were used to represent quantitative data. To assess the relationship between qualitative variables, the Chi-squared test (χ^2^) was used. For small-frequency distributions, Fisher's exact test was used. The Mann-Whitney test was used to find the relationship between non-parametric variables in quantitative data. The odds ratio was calculated at a confidence interval (CI) of 95% when doing the multivariate logistic regression analysis to assess the risk factors (independent predictors) of IBS. The R-squared (R² or coefficient of determination) was determined, which is a statistical measure in a regression model that determines the proportion of variance in the dependent variable that can be explained by the independent variable. R-squared values range from 0 to 1. A value of 0 implies that the independent variable(s) has no explanatory power, while a value of 1 indicates a perfect. The R2 value of the regression model was 0.62, which suggests that 62% of IBS cases can be explained by the included variables. A p-value of less than 0.05 was considered statistically significant.

## Results

This study involved a total of 335 participants whose mean age was 28.65 ± 8.01 years, and 56.1% were female. Of them, 71.1% had a university-level education, 56.4% were unmarried, 47.5% were working, and 17% were smokers.

Of the studied participants, 28.4% were previously diagnosed with IBS. About 57% (57.6%) suffered frequent abdominal pain in recent months, and of them, 58.1% reported that pain increased or decreased with defecation. Almost one-third (29.9%) noticed a change in the nature of the stool, and 34.9% had a change in the frequency with which they empty the bowels. The majority (79.1%) did not use any medications to treat colon symptoms. About half (47.5%) reported that pain increases with eating, and for 78.6%, pain increases with spicy food (Table 1).

**Table 1:** Distribution of studied participants according to previous diagnosis with IBS, its symptoms, treatment, effect of pain, and family history of IBS (N:335) IBS: irritable bowel syndrome

Variable	N.			%
Have you ever been diagnosed with irritable bowel syndrome?				
No	240		71.6	
Yes	95		28.4	
Do you suffer from frequent abdominal pain (once or more) in recent months?				
No	142	42.4		
Yes	193	57.6		
Is the pain increased or decreased with defecation (empty of bowels)? (N:193)				
No	81	41.9		
Yes	112	58.1		
Have you noticed any change in the stool shape?				
No	235	70.1		
Yes	100	29.9		
Have you noticed any change in the number of times to go to the toilet to empty the bowels?				
No	218	65.1		
Yes	117	34.9		
How many types of medications do you regularly use to treat colon symptoms?				
One	26	7.8		
Two	22	6.6		
Three	10	3.0		
Four	8	2.4		
More than five	4	1.2		
None	265	79.1		
Does abdominal pain affect your social and educational level?				
No	256	76.4		
Yes	79	23.6		
Does the pain increase with eating?				
No	176		52.5	
Yes	159		47.5	
If yes, which of the following foods increases pain? (N:159)				
Dairy products (milk, cheese, etc.)	65		40.8	
Fatty foods	110		69.1	
Spicy foods	125		78.6	
Wheat (bread, cereals, etc.)	45		28.3	
Sugar and substitutes	18		11.3	
Is there any member of the family suffering from irritable bowel syndrome?				
No	149		44.5	
Yes	186		55.5	

According to the Rome IV criteria, the prevalence of irritable bowel syndrome among the studied population represented 30.4% (n = 102). Generalized anxiety disorder was detected among 53 (15.8%) of the participants, which was subdivided into 35 (10.4%) moderate anxiety and 18 (5.4%) severe anxiety disorder.

Irritable bowel syndrome was significantly higher among single participants with university-level education, workers, non-smokers, and those having a family history of IBS, with p-values of <0.001, 0.023, 0.006, 0.016, and < 0.001, respectively (Table 2).

**Table 2:** Relationship between IBS prevalence and participants' demographic characteristics, smoking status, and family history of IBS (N:335) IBS: irritable bowel syndrome; p-value less than 0.05 is statistically significant; * Fischer's exact test

Variable	IBS N. (%)	No IBS N. (%)	χ^2^	p-value
Age	28.7 ± 7.6	28.63 ± 8.2	0.25	0.798
Gender				
Female	57 (30.3)	131 (69.6)	0.003	0.954
Male	45 (30.6)	102 (69.4)		
Marital status				
Widow	0 (0.0)	3 (100.0)	16.47*	<0.001
Single	47 (24.9)	142 (75.1)		
Married	45 (34.6)	85 (65.4)		
Divorced	10 (76.9)	3 (23.1)		
Educational level				
Primary school	2 (66.7)	1 (33.3)	11.68*	0.012
Middle school	1 (16.7)	5 (83.3)		
Secondary school	23 (41.8)	32 (58.2)		
University education	62 (25.8)	178 (74.2)		
Postgraduate education	14 (45.2)	17 (54.8)		
Occupation status				
University students	37 (38.1%)	60 (61.9%)	10.39	0.006
Working	52 (32.7)	107 (67.3%)		
Non-working	13 (16.5%)	66 (83.5)		
Smoking				
No	77 (27.7)	201 (72.0)	5.83	0.016
Yes	25 (43.9)	32 (56.1)		
Is there any member of the family suffering from irritable bowel syndrome?				
No	20 (13.4)	129 (86.6)	36.73	<0.001
Yes	82 (44.1)	104 (55.9)		

**Figure 1:**
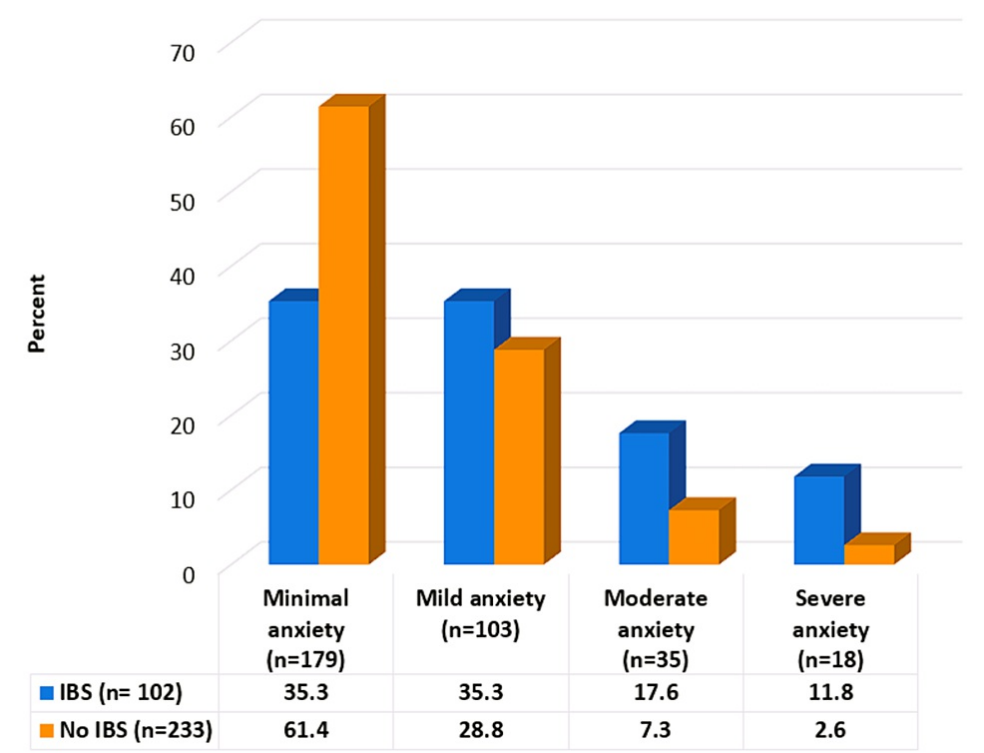
Relationship between IBS prevalence and GAD subtypes IBS: irritable bowel syndrome; GAD: generalized anxiety disorder; χ^2^ = 28.44, p<0.001

Irritable bowel syndrome was significantly higher among participants who had generalized anxiety disorder (p<0.001) (Figure 1).

A multivariate logistic regression analysis was done to assess the risk factors for IBS. The regression model revealed that being unmarried, having a family history of IBS, and having GAD were risk factors for developing IBS among the studied participants (Table 3).

**Table 3:** Multivariate logistic regression analysis of risk factors for IBS among studied participants IBS: irritable bowel syndrome; GAD: generalized anxiety disorder; p-value less than 0.05 is statistically significant.

Variable	B	Wald	p-value	Odds Ratio (CI:95%)
Marital status	1.84	12.25	<0.001	1.19 (2.23-4.67)
Educational level	0.35	2.75	0.097	1.42 (0.93-1.17)
Smoking	0.8	1.3	0.21	0.44 (0.22- 0.88)
Family history of IBS	1.59	26.94	<0.001	1.43 (2.17-5.36)
Having GAD	6.44	17.9	<0.001	1.47 (2.87-4.15)

## Discussion

Irritable bowel syndrome is a prevalent medical disorder that decreases a patient's quality of life. Despite research on the global frequency and etiology of IBS, there is insufficient evidence of its prevalence and risk factors in Saudi Arabia's general population. So, the purpose of this study was to investigate the prevalence and risk factors of irritable bowel syndrome in individuals in Saudi Arabia's Al-Qunfudah governorate.

In the present study, the majority of study subjects suffered from frequent abdominal pain (once or more) in recent months (57.6%), the pain increased or decreased with defecation (58.4%), and they did not notice any change in the stool shape (70.1%) or the frequency to empty the bowels (65.2%), and the pain could increase with some eating spicy and fatty food (78.1%, 69.1%, respectively), in agreement with Khan et al. [19]. Furthermore, this study revealed that adult IBS prevalence was 30.4%, which is relatively higher than that reported in previously done Saudi studies, which recorded a prevalence of 17-24% [7, 20-22]. Without a doubt, the prevalence of IBS varied between studies due to a range of factors such as research population, study process, and sociocultural differences.

Generalized anxiety disorder was discovered among 15.8% of the study sample, which is similar to 12.7% of another Saudi study [23]. This high prevalence of generalized anxiety disorder among the adult general population in Al-Qunfudah governorate may be due to the fact that most of the study participants were students and working adults, and there are many previous Saudi studies that concluded that anxiety is common among students and working adults [22-24]. Stress alters gut physiology by increasing visceral perception, intestinal permeability, modifications in gastrointestinal secretion, negative effects on mucosal blood flow, and gastrointestinal mucosa regeneration capacity [25]. This hypothesis can explain our finding of a significant association between IBS and generalized anxiety disorder (GAD), as 29.4% of participants with anxiety disorder (GAD) were suffering from IBS symptoms (p<0.001). These findings are consistent with those of Modabbernia et al. [26], who found a significant connection between IBS and GAD. The ability of healthcare providers to recognize and treat these co-morbidities could improve patient outcomes.

The study found that females (56.1%) had a greater prevalence of IBS than males (43.9%), with no significant relationship. This finding is in accordance with the results of many studies [27-30]. In any case, it is uncertain what causes gender variations in IBS [31]. However, according to Anbardan et al. [32], female dominance is due to the fluctuation of reproductive hormones and their impact on intestine mortality. Furthermore, stress encountered during the menstrual cycle makes IBS symptoms worse. Smoking showed no association with IBS disease, which is corroborated by Sirri et al.'s [33] systematic review. This conclusion contrasts with a study among Makkah citizens in which smokers (23.1%) were more likely to have IBS [22], and the difference between our and their results could be due to differences in sample size and the characteristics of both study groups.

This study revealed that risk factors for developing IBS were being single, having an anxiety disorder, or having a family history of IBS. The familial risk factor for IBS was approved by previous Saudi studies [34, 35]. Additionally, Eijsbouts et al. [36] explained the familial nature of IBS by discovering six genetic loci for IBS susceptibility (BAG6, CKAP2/TPTE2P3, DOCK9, NCAM1, CADM2, and PHF2/FAM120A) and found that the first four genes are also linked to mood and anxiety disorders, which makes GAD and IBS closely related. This study detected that being unmarried is a risk factor for developing IBS, which is contradicted by a previous Saudi study that found an association between IBS and being married [37].

Limitations

One of the challenges of the study is that the questionnaire was completed online instead of in an interactive interview. Another is that most of the participants were young adults who were literate, had access to the internet, and knew how to use internet-based technologies; therefore, the other techno-illiterate adults were denied and not examined. Selection bias, a risk associated with choosing the study sample using a non-probability technique, is another constraint of this research. Furthermore, causality cannot be proved because of the cross-sectional nature of this study's methodology. A prospective study would be necessary to demonstrate causation. Notwithstanding these limitations, the purpose of this study is to increase public awareness of this common disorder.

## Conclusions

In the Al-Qunfudah governorate, the prevalence of IBS is 30.4% among adults, while 15.8% of the participants suffered from generalized anxiety disorder. There is an association between anxiety and IBS, as 29.7% of those with IBS suffer from generalized anxiety disorder. Risk factors for IBS were being unmarried, having anxiety, and having a family history of IBS. We advise launching stress-coping technique initiatives in the Al-Qunfudah governorate. It is also necessary to educate people about the signs and consequences of this disorder. To control the severity of this problem and its impact on a person's quality of life, we also recommend that those who are suffering from IBS symptoms obtain a second opinion from a doctor. It is advised to conduct more prospective studies with careful attention to confounders to identify the underlying etiologies of this disease. Future research should be done with larger samples, as well as assessing procedures aimed at addressing the prevalence of IBS and psychological co-morbidities in the population. An interactive interview-based study can be more helpful in estimating the prevalence of IBS and GAD among adults to ensure the participation of non-educated participants and those who cannot use technology to answer online surveys. We recommend applying this study and selecting participants using a simple random sampling approach to overcome the bias of the non-probability sampling technique. Prospective research is recommended to establish causation.
